# Case of Injury of the Brain, and Loss of Substance; with Recovery

**Published:** 1854-11

**Authors:** B. Woodward


ART. IV.—*Case of Injury of the Brain, and Loss of Substance; with Recovery.* By B. WOODWARD, M.D.

On the evening of the 5th of August last I was called to see a boy 6 years old, who had been kicked by a colt. The injury had occurred some four hours previous to my seeing him. The child was insensible. The whole upper part of the nose was broken down, and the bones driven over under the integuments of the left cheek, under the eye. On cleansing the wound, I found not only the nose broken down, but the skull fractured over the right eye, and under the eye-brow, and a mass of brain (lacerated) as large as a large hickory nut protruding. As soon as efforts were made to replace the bones of the nose, violent vomiting was produced, and more of the brain was forced out. I deemed it best to call council, and Doctor Porter of Prophetstown was called in. On making renewed efforts to replace the bones of the nose, he found the vomiting and consequent protrusion of the brain so great as to cause him to desist; and we concluded to meddle with the nose as little as possible, for fear of still further endangering the life of the child. The nasal passages were cleared, the brain, which had exuded, removed, and the wound dressed.

The next day, the father of the child, not being satisfied with his looks, Doctor Cottle of Albany was sent for. He thought, as we had done, that it was best to meddle with the nose as little as possible, as there seemed already but little chance for the life of the child.

The case was now left in my hands; but I would here acknowledge my obligations to both the medical gentlemen for their valuable advice as to the further management of the case.

For eight days there were more portions of the brain protruded, which I as often removed, and trusted entirely to keeping the head cool, the bowels open with saline purgatives, and a low diet, except that I used the nit. argent. freely to the broken or cut surface of the brain. I tried compresses from time to time, in order to prevent the loss of the substance of the brain; but the least pressure would produce convulsions. I would only state further, that in due time the wounds all healed, and the boy recovered.

This case has taught me never to despair as long as there is life.

